# Relationship between decreased cerebral blood flow and amnesia after microsurgery for anterior communicating artery aneurysm

**DOI:** 10.1007/s12149-020-01436-z

**Published:** 2020-01-27

**Authors:** Shunji Mugikura, Naoko Mori, Hirokazu Kikuchi, Etsuro Mori, Shoki Takahashi, Kei Takase

**Affiliations:** 1 Department of Diagnostic Radiology, Tohoku University Graduate School of Medicine, 1-1 Seiryo-machi, Aoba-ku, Sendai, 980-8574 Japan; 2 Department of Neurology, Tohoku Medical and Pharmaceutical University, Sendai, Japan; 3 Department of Behavioral Neurology and Neuropsychiatry, Osaka University United Graduate School of Child Development, Suita, Japan

**Keywords:** Cerebral blood flow, Amnesia, Aneurysm, SPECT and neuropsychologic tests

## Abstract

**Objective:**

Postoperative amnesia after surgery for anterior communicating artery aneurysm might be associated with the damage of the basal forebrain. Our purpose was to verify whether decreased regional cerebral blood flow (rCBF) in basal forebrain could be related to the degree of postoperative amnesia.

**Methods:**

Regional voxel rCBF data analyzed using three-dimensional stereotactic surface projection on ^123^I-IMP-SPECT were compared between ten patients with postoperative amnesia and 13 normal subjects. The Severity (average *Z* score of the voxels with a *Z* score that exceeds a threshold value of 2) was calculated. The cerebral lobes with rCBF exhibiting Severity > 2 in all patients were identified. In these lobes, we then examined whether there was a gyrus exhibiting Severity that was negatively related to memory quotients (MQs).

**Results:**

In the right subcallosal gyrus, there was a significant negative correlation between Severity and visual MQ (*ρ*= −  0.884, *p* = 0.0007) or general MQ (*ρ* =− 0.853, *p* = 0.0017). In the right anterior cingulate gyrus, there was a significant negative correlation between Severity and verbal MQ (*ρ* = − 0.769, *p* = 0.0092). In the right rectal gyrus, there was a significant negative correlation between Severity and general MQ (*ρ* =  −  0.811, *p* = 0.0044). No significant correlations were found between Severity in other brain regions and verbal, visual, or general MQ.

**Conclusions:**

The decreased rCBF in the subcallosal gyrus included in the basal forebrain, anterior cingulate gyrus, and the rectal gyrus in the right hemisphere was related to postoperative amnesia.

**Electronic supplementary material:**

The online version of this article (10.1007/s12149-020-01436-z) contains supplementary material, which is available to authorized users.

## Introduction

Postoperative amnesia or memory impairment after surgery for anterior communicating artery (ACoA) aneurysm has been repeatedly reported since the 1950s, and is known as “ACoA syndrome” [1]. Postoperative amnesia can severely affect patients’ quality of life [2].

In 1986, Damasio et al. hypothesized that postoperative amnesia might be related to damage of the basal forebrain in the ventral and medial region of the cerebral hemisphere, caused by aneurysmal rupture or by the surgical procedure [3]. In the hypothesis, the basal forebrain is considered to play an important role in memory, because it includes the cholinergic nuclei that project to the hippocampus and cerebral hemisphere [3]. Therefore, postoperative amnesia is often referred to as basal forebrain amnesia. However, this hypothesis has never been validated yet.

Previous studies have found that the basal forebrain is mainly perfused by the subcallosal artery, the largest unpaired perforating artery of the AcoA [4, 5]. The subcallosal artery is likely to be occluded in association with aneurysmal rupture or surgical procedures, because it originates closely to the ACoA aneurysm. Recent reports using magnetic resonance (MR) imaging have suggested that postoperative amnesia could be related to occlusion of the subcallosal artery with resultant infarcts in the territory of the subcallosal artery [6, 7]. Therefore, the damage of the basal forebrain might be the mixture of factors such as infarcts or various degree of ischemia in the subcallosal artery territory and direct damage by aneurysmal rupture or surgical procedure.

To evaluate the degree of the damage of basal forebrain, assessment of cerebral blood flow of basal forebrain would be needed rather than MR imaging. Single photon emission computed tomography (SPECT) is generally used to evaluate the regional cerebral blood flow (rCBF) in patients with memory impairment. In patients with memory impairment after surgery for ACoA aneurysm, there might be a different pattern or degree of rCBF changes in the basal forebrain possibly due to the mixture of factors such as infarcts or various degree of ischemia in the subcallosal artery territory and direct damage by aneurysmal rupture or surgical procedure.

Thus, we hypothesized that examination of the relationship between decreased rCBF and memory impairment could provide a clue to the mechanisms of postoperative amnesia. Specifically, we focused on examining whether decreased rCBF in basal forebrain could be related to the degree of memory impairment in patients with postoperative amnesia.

## Materials and methods

### Patients

Our institutional review board approved this retrospective study with a prospective data collection, and written informed consent was waived. From December 2007 to March 2013, 14 consecutive patients with suspected postoperative amnesia visited the behavioral neurology department of our hospital. Patients suspected of postoperative amnesia were evaluated with formal neuropsychological examinations, including the Wechsler Adult Intelligence Scale III (WAIS-III) [8] and Wechsler Memory Scale-Revised (WMS-R) [9], SPECT and MR imaging.

After ACoA aneurysm surgery, associated intraparenchymal hematoma, hydrocephalus, and vasospasm have all been reported to lead to deterioration of memory as well as overall intelligence [1, 10]. To clarify the relationship of SPECT findings specifically with the degree of memory impairment but not with overall impairment of intelligence, our inclusion criteria to select patients were those exhibiting memory quotient and a difference ≥ 15 between the full-scale intelligence quotient (FSIQ) on the WAIS-III and the general memory quotient (GMQ) on the WMS-R (FSIQ-GMQ) [7]. Thus, we included only patients who showed a specific impairment in memory. Of those included, eight patients had ruptured ACoA aneurysms and the remaining two had unruptured ACoA aneurysms. Patient demographic data are shown in Table 1.

**Table 1:** Patient demographic data and summary of neuropsychological findings

Patient	1	2	3	4	5	6	7	8	9	10
Age (year)	52	42	39	45	54	45	69	55	39	59
Sex	M	M	M	M	M	M	M	M	M	F
Ruptured (R)/ unruptured (U)	R	R	R	R	R	R	U	R	R	U
Treatment	Trap	Clip	Clip 2nd	Clip	Clip	Clip	Clip	Clip	Clip	Clip
Months from treatment	2	4	4*	3	13	3	3	5	3	4
MMSE	24	25	26	28	25	28	26	24	23	27
Full-scale IQ	82	97	111	110	120	106	102	83	89	92
Verbal MQ	62	57	65	91	93	85	88	63	66	75
Visual MQ	69	81	90	98	93	98	86	81	87	64
General MQ	58	59	68	92	92	87	86	64	68	67
Attention/ concentration	81	80	114	138	131	115	115	91	94	133
Delayed recall	< 50	< 50	< 50	< 50	64	73	62	< 50	< 50	< 50
Full-scale IQ minus general MQ	24	38	43	18	28	19	16	19	21	25

4* in the row of “months from treatment” indicates 4 months after second clipping. MMSE, used to assess cognitive impairment (full score 30); Full-scale IQ, full-scale intelligence quotient evaluated by Wechsler Adult Intelligence Scale III (WAIS-III) [8]; General MQ; memory quotient, Attention/concentration quotient, and Delayed recall quotient evaluated by Wechsler Memory Scale-Revised (WMS-R) [9]. Each quotient has a mean of 100 in the normal population and a standard deviation of 15. A substantial difference between Full-scale IQ and General MQ in all patients; Full-scale IQ minus General MQ > 15, which indicates that the patient with amnesia has a particular impairment in memory but not in the “intelligence” per se [7]

Formal neuropsychological examinations and SPECT were performed a median of 4 months (range 2–13 months) after ACoA aneurysm surgery, within 1 month of each other. This delayed timing of formal neuropsychological examinations after surgery was considered desirable because assessment in the acute phase (from 1 to 6 weeks) after aneurysm surgery would be unreliable [11]. In the acute phase, patients exhibit a state of confusion with disorientation and intellectual disturbance, and accurate diagnosis of amnesia is difficult.

### Neuropsychological assessment

We administered a set of formal neuropsychological examinations including the WAIS-III as an examination of overall intelligence [8] and the WMS-R as an examination of anterograde memory [9]. Using the WAIS-III, full-scale IQ can be quantified. The WMS-R is a standardized neuropsychological examination of anterograde memory and five scores (verbal MQ, visual MQ, general MQ, attention/concentration, and delayed recall) can be quantified.

### SPECT imaging

SPECT was performed in chronic phase (range 2–13 months) after ACoA aneurysm surgery within 1 month of formal neuropsychological examinations.

Two SPECT scanners (Multi-SPECT3, Siemens Medical Systems, Munich, Germany and PRISM-IRIX; Shimadzu Co, Tokyo, Japan) with a three-head rotating gamma camera were used for all SPECT studies. Each SPECT imaging scan was started in the resting state, 15 min after intravenous bolus injection of 111 MBq (3 mCi) of 123 N-isopropyl-*p*-iodoamphetamine (^123^I-IMP). The fitted collimator was low-energy, high-resolution, and in-plane, with axial resolutions of 10.6 mm full width at half maximum. Image reconstruction was performed by filtered back projection with a Butterworth filter, and attenuation correction was performed numerically by assuming the object shape to be an ellipse for each slice and the attenuation coefficient to be uniform (0.08 per cm). For Multi-SPECT3, projection data were acquired in a 128 × 128 format for 30 projections at 50 s with 120° rotation of the camera. For PRISM-IRIX, projection data were acquired in a 128 × 128 format for 37 projections at 40 s with 120° rotation of the camera. No quantitative analyses for determination of CBF were performed on all SPECT data.

### SPECT analysis using 3D-SSP

3D-stereotactic surface projection (SSP) was performed using a graphical user interface, Stereotactic Surface Projections (iSSP), installed in NEUROSTAT (AZE Ltd, Tokyo, Japan) [12]. Stereotactic anatomical standardization was performed by transforming the original 123I-IMP SPECT images into standard Talairach space. Differences in size between the individual brains and the standard template were removed by linear scaling. Regional anatomical differences between the individuals and the standard template were minimized by automated non-linear warping. The peak cortical activity in the brain was then subjected to a 3D search with a predefined vector, which was a depth of six pixels (13.5 mm) in the vertical direction in the cortex, for each stereotactic surface pixel after anatomical standardization. The peak value was projected back and assigned to the originating surface pixel. This procedure was continued on a pixel-by-pixel basis covering the whole cortex of the brain. The voxel values of an individual’s image set were normalized to the whole brain tracer uptake. After comparing the CBF of patients to that of the 13 age-matched normal databases by each voxel, the abnormalities of cerebral hypoperfusion were displayed with a *Z *score map. *Z *scores were calculated using the following equation: *Z *score = (normal mean-patient mean)/(normal standard deviation). We used a *Z *score of 2 as the cut-off value in each voxel, and voxels with a Z score ≤ 2 were considered voxels without significantly decreased rCBF. The Severity (average *Z* score of voxels with a *Z* score exceeding the threshold value of 2) and the Extent of an abnormal region in each segment (percentage of voxels with a *Z* score exceeding the threshold value of 2 within a segment) were calculated [13].

Severity was evaluated first with the level 2 analysis of 3D-SSP, in which the calculation was made according to ten cerebral lobes in both hemispheres (frontal, parietal, temporal, limbic, and occipital). Since the lobes that did not have significantly decreased rCBF in all patients were considered to be not prerequisite for memory impairment in postoperative amnesia, we identified the lobes with Severity > 2 in all patients in common and regarded them as lobes with decreased rCBF related to memory impairment.

Next, Severity and Extent were evaluated with level 3 analysis of 3D-SSP to find the gyri or regions related to memory impairment: Of the 64 subdivided regions of the ten cerebral lobes in 3D-SSP, both Severity and Extent were calculated for the subdivided regions of the lobes with substantially decreased rCBF in the afore-mentioned level 2 analysis of 3D-SSP.

### Analysis and statistics

We examined the relationship between the Severity or Extent on SPECT and neuropsychological assessment scores by WMS-R using Spearman’s rank correlation. A *p* value less than 0.01 was considered significant.

## Results

The neuropsychological findings are summarized in Table 1. WMS-R examination could be performed appropriately, because Attention and Concentration in all patients were higher than 80. However, delayed recall, representing the maintenance of memory after 30 min, was below the measurable limit (< 50) in seven patients, and therefore, the relationship between the Severity or Extent on SPECT and delayed recall was not examined.

**Fig. 1:**
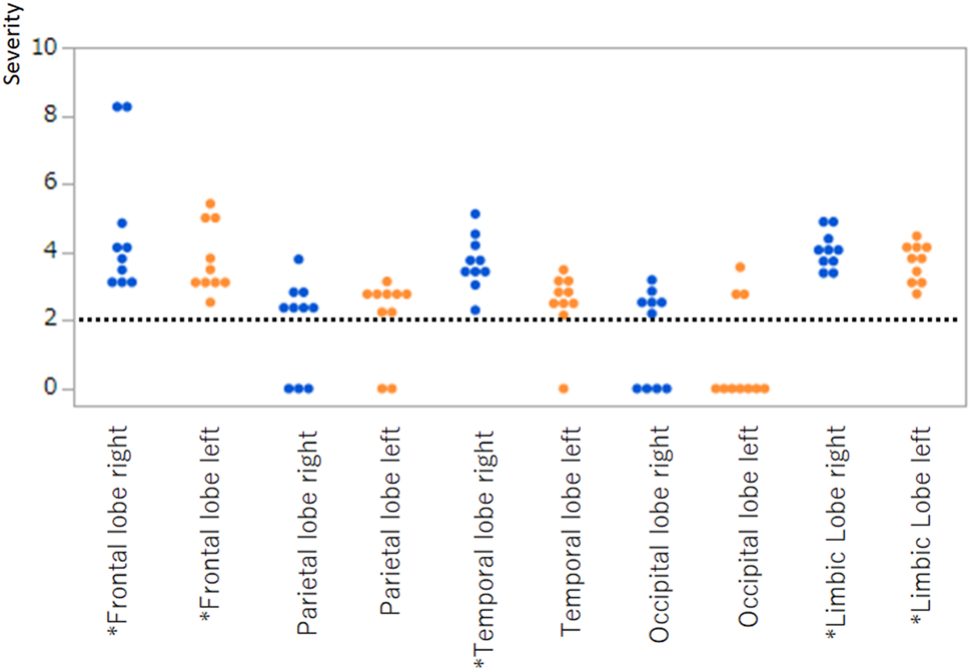
Severity of decreased rCBF in five cerebral lobes in each hemisphere (frontal, parietal, temporal, limbic, and occipital) in each patient. Each dot indicates the Severity of decreased rCBF in each patient. *Indicates the lobes in which the Severity of decreased rCBF > 2 in all patients in common

In the level 2 analysis, the lobes exhibiting Severity of decreased rCBF > 2 in all patients in common were the bilateral frontal and limbic lobes, and the right temporal lobe (Fig. 1).

In the level 3 analysis, Severity and Extent of subdivided gyri within bilateral frontal, bilateral limbic, and right temporal lobes, which revealed to have substantially decreased rCBF in level 2 analysis, were calculated. For subdivisions prepared in the level 3 analysis of 3D-SSP, the frontal lobe has nine subdivisions (superior frontal gyrus, middle frontal gyrus, inferior frontal gyrus, medial frontal gyrus, orbital gyrus, rectal gyrus, paracentral lobule, precentral gyrus, and subcallosal gyrus), the limbic lobe has 8 subdivisions (fusiform gyrus, lingual gyrus, thalamus, cingulate gyrus, parahippocampal gyrus, anterior cingulate, posterior cingulate, and uncus) and the temporal lobe has four subdivisions (superior temporal gyrus, middle temporal gyrus, inferior temporal gyrus, and transverse temporal gyrus)(appendix). Since 3D-SSP has only subcallosal gyrus for the basal forebrain (that may additionally include the paraterminal gyrus, diagonal band of Broca, substantia innominata, and nucleus accumbens) [14], we used rCBF of the subcallosal gyrus for rCBF of the basal forebrain in this study.

**Fig. 2:**
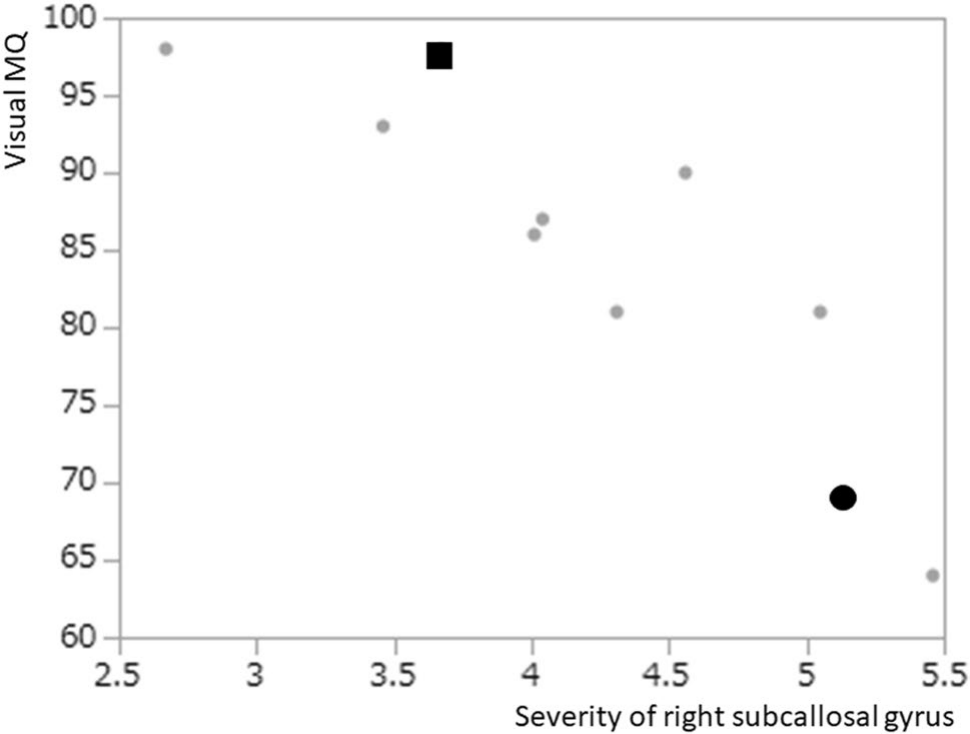
The relationship between the *Severity* of decreased rCBF in the right subcallosal gyrus and the visual MQ by the WMS-R in each patient (*ρ* =  − 0.88, *p* = 0.0007). The large circular dot indicates the plot of the patient shown in Fig. 3 (patient 1). The square dot indicates the plot of the patient shown in Fig. 4 (patient 4)

**Fig. 3:**
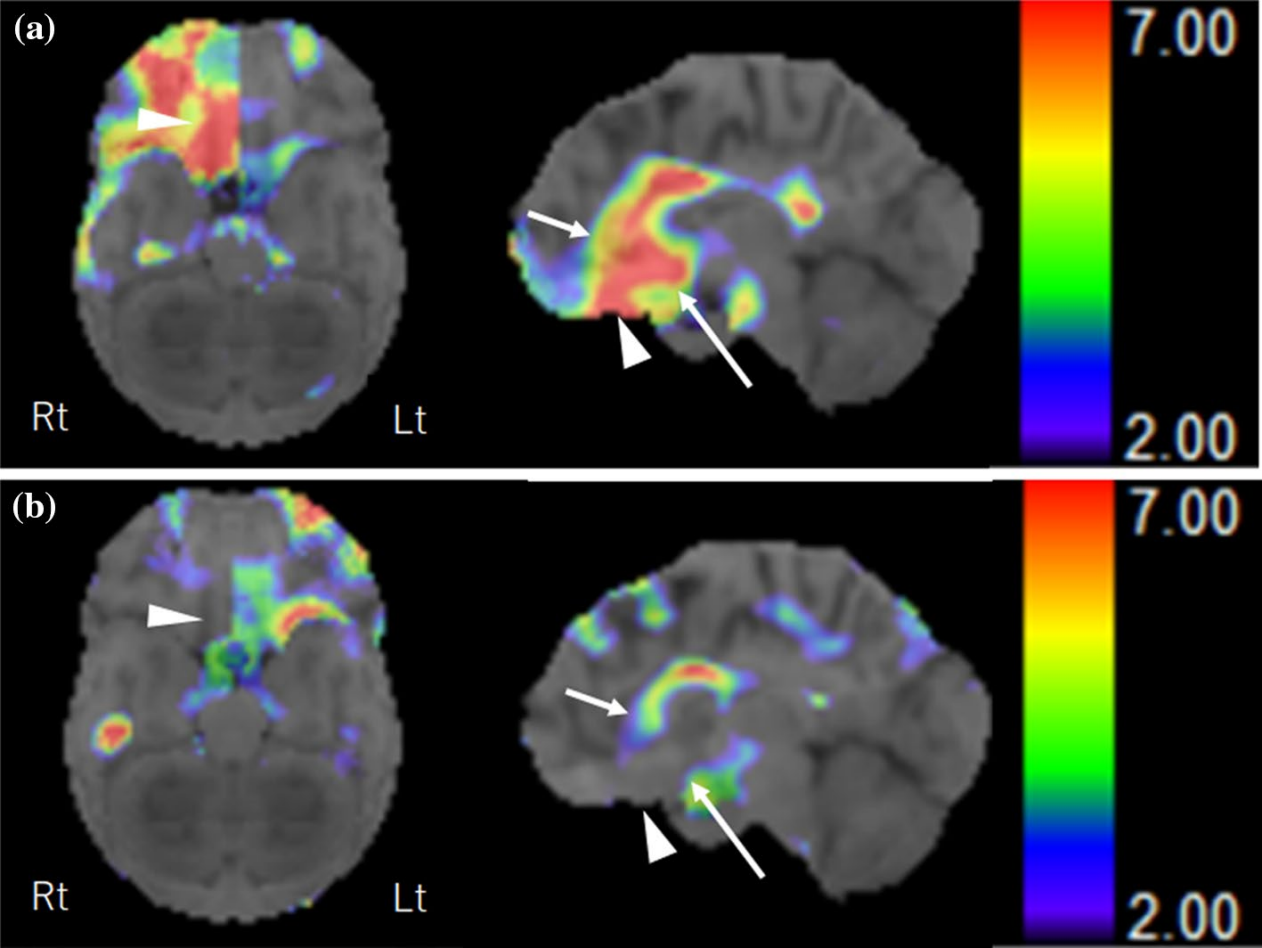
Stereotactic extraction estimation of decreased rCBF showing *Z* score. The left panel shows the inferior view and the right shows the medial view of the right hemisphere. The color scale bar shows the *Z* score ranging from 2 to 7. **a** Patient 1: a 52-year-old man presented with ruptured aneurysm of ACoA. Surgical clipping for the aneurysm was performed on the day of onset. Formal neuropsychological examination and SPECT were performed 2 months after the treatment of aneurysm. High Severity of decreased rCBF was observed in the subcallosal gyrus (5.13, arrow), anterior cingulate gyrus (5.78, short arrow), and rectal gyrus (7.99, arrowhead) on the right. All three MQs on the WMS-R were severely decreased (Verbal MQ: 62, Visual MQ: 69, and General MQ: 58). **b** Patient 4: a 45-year-old man presented with ruptured aneurysm of ACoA. Surgical trapping of ACoA for the ruptured ACoA aneurysm was performed the day of onset. Formal neuropsychological examination and SPECT were performed 3 months after the treatment of aneurysm. Low Severity of decreased rCBF was observed in the subcallosal gyrus (3.67, arrow), anterior cingulate gyrus (3.42, short arrow), and rectal gyrus (2.33, arrowhead) on the right. All three MQs on the WMS-R were preserved (Verbal MQ: 91, Visual MQ: 98, and General MQ: 92)

Of the subdivided gyri examined, the following gyri showed significant correlation with MQs. In bilateral frontal lobes, Severity of right subcallosal gyrus had a significant negative correlation with visual MQ (*ρ* = − 0.884, *p* = 0.0007) and general MQ (*ρ* = − 0.853, *p* = 0.0017) (Table 2a, Fig. 2). Severity in the right rectal gyrus had a significant negative correlation with general MQ (*ρ* = − 0.811, *p* = 0.0044) (Table 2a). Patients with higher *Severity* of decreased rCBF in subcallosal (Figs. 2, 3a) or rectal gyri on the right exhibited lower MQs, while patients with lower Severity in these gyri showed relatively higher MQs (Figs. 2, 3b).

**Table 2:** Relationship between decreased rCBF in level 3 analysis of 3D-SSP and memory quotients (MQs)

(a)	*Z* score (median, 25, 75 percentile)	Verbal MQ	Visual MQ (*ρ*)	General MQ (*ρ*)
Subcallosal gyrus R	4.16 (3.62, 5.07)		*ρ = −* 0.884 *p * = 0.0007	*ρ = −* 0.853 *p *= 0.0017
Rectal gyrus R	5.36 (2.94, 7.22)			*ρ = −* 0.811 *p *= 0.0044
Anterior cingulate gyrus R	4.44 (3.85, 4.91)	*ρ = −* 0.769 *p * = 0.0092		

(b)	Percent (median, 25, 75 percentile)	Verbal MQ	Visual MQ (*ρ*)	General MQ
Subcallosal gyrus R	85 (45, 100)		*ρ = −* 0.770 *p *= 0.0091	

(a) Areas or gyri in which the Severity of the decreased rCBF was significantly related to three MQs in the Wechsler Memory Scale-Revised (WMS-R)

(b) Areas or gyri in which the Extent of decreased rCBF was significantly related to three MQs in the Wechsler Memory Scale-Revised (WMS-R)

In the bilateral limbic lobes, Severity of the right anterior cingulate gyrus had a significant negative correlation with verbal MQ (*ρ* = − 0.769, *p* = 0.0092) (Table 2a). Patients with higher Severity of decreased rCBF in the right anterior cingulate gyrus exhibited lower general MQ, while patients with lower Severity exhibited relatively higher general MQ (Fig. 3a and b). Severity in any other gyri in frontal or limbic lobes showed no significant correlation with MQs (appendix). Any gyri of the right temporal lobe had no significant correlation between Severity and MQs.

Regarding the *Extent* of the gyri, only the right subcallosal gyrus had a significant negative correlation with visual MQ (*ρ* =  −  0.770, *p* = 0.0091) (Table 2b). Any other gyri in bilateral frontal, limbic, or right temporal lobes had no significant correlation between Extent and the evaluated items of MQs.

## Discussion

The current study revealed that the decreased rCBF in the subcallosal gyrus (basal forebrain), anterior cingulate gyrus, and rectal gyrus, especially in the right hemisphere, were related to postoperative amnesia. To our knowledge, this is the first study to examine the relationship between the rCBF by SPECT and memory impairment by WMS-R in the same period after ACoA aneurysm surgery.

In level 2 analysis of 3D-SSP, the current study revealed that rCBF in the frontal and limbic lobes on both sides and the temporal lobe on the right was significantly decreased than those of normal database in all 10 patients in common. Severity of decreased rCBF in the frontal lobe on both sides might be related to the location of the ACoA aneurysm in the most ventral part of the circle of Willis. Decreased rCBF in both frontal lobes as shown in our study might be related to neuropsychological frontal lobe dysfunction that is frequently observed in cases with ruptured ACoA aneurysm [1]. Meanwhile, the Severity of decreased rCBF in the right temporal and bilateral limbic lobes might be related to the hypofunction of the Papez memory circuit included in these lobes. Indeed, the previous studies reported the high prevalence of infarcts in the column of the fornix (FxCo) and anterior cingulate gyrus, constituent parts of the Papez memory circuit, after ACoA aneurysm surgery [6, 7].

In level 3 analysis of 3D-SSP, the Severity of decreased rCBF in the subcallosal gyrus, rectal gyrus, or anterior cingulate gyrus all on the right was significantly related to at least one of the three MQs. The Extent of decreased rCBF in the right subcallosal gyrus was significantly related to the decrease in visual MQ.

**Fig. 4:**
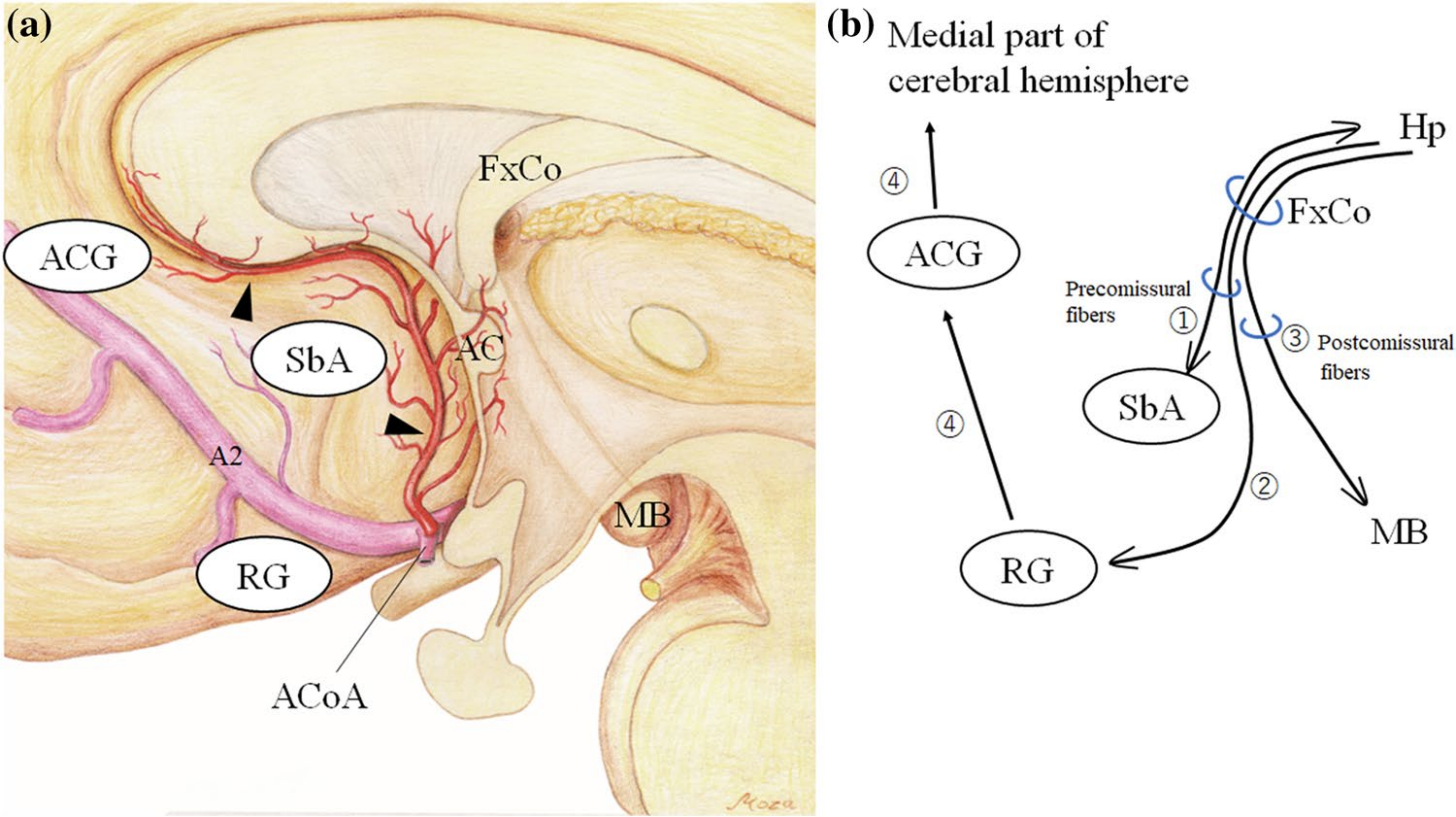
**a** An illustration of the subcallosal artery (arrowheads) supplying the ventral and medial region of the cerebral hemisphere on the basis of a previous study regarding the microsurgical anatomy of the artery [4] (reproduced from Mugikura et al. [7]). The subcallosal artery originates from the posterosuperior aspect of the ACoA, ascends dorsally into the lamina terminalis cistern, and then curves forward and upward. The artery supplies regions including the subcallosal gyrus (SbA), anterior commissure (AC), column of fornix (FxCo), and anterior cingulate gyrus (ACG). MB, mammillary body; A2, A2 segment of the anterior cerebral artery; RG, rectal gyrus. **b** An illustration of the relationship among SbA, RG, and ACG, where the Severity of decreased rCBF was significantly related to Memory Quotients (MQs) by WMS-R. The cholinergic nuclei in the SbA project the cholinergic fibers to the hippocampus (Hp) through the FxCo (①). RG receives input fibers from Hp through the FxCo (②). The FxCo is a part of the Papez memory circuit connecting the Hp and MB (③). RG projects cholinergic fibers through CGa to the medial part of the cerebral hemisphere (the medial cholinergic pathways, ④). ① and ② run through the precommissural fibers and ③ runs through the postcommissural fibers

The cholinergic nuclei in the subcallosal gyrus project cholinergic fibers to the hippocampus through the FxCo (Fig. 4) and this projection plays a crucial role in the modulation of memory [11]. The relationship between decreased rCBF in the subcallosal gyrus and memory impairment should support the hypothesis regarding a causal relationship between the subcallosal gyrus and postoperative amnesia [3]. Receiving input fibers from the hippocampus through the FxCo (Fig. 4) [15], the rectal gyrus projects cholinergic fibers to the anterior cingulate gyrus, a part of the Papez memory circuit, finally reaching the medial part of the cerebral cortex (the medial cholinergic pathways) (Fig. 4) [16]. Therefore, decreased rCBF in the rectal and subcallosal gyri might also be related to memory impairment.

The following mechanism was considered as the cause of decrease of rCBF in the subcallosal, rectal, and anterior cingulate gyri. Both subcallosal and anterior cingulate gyri are known to be perfused by the unpaired subcallosal artery from the ACoA [4, 5], and the previous studies reported that infarction caused by the occlusion of the subcallosal artery was related to postoperative amnesia [6, 7]. Thus, the current results indicate that decreased rCBF found in both subcallosal and anterior cingulate gyri could be the results of injury or occlusion of that artery in the perioperative period.

Regarding the rectal gyrus, its damage might have been caused by aneurysmal rupture or surgical procedure [17, 18]. Furthermore, infarcts in the rectal gyrus might be associated with the occlusion of the recurrent artery of Heubner, which usually originates on both sides around the junction of the ACoA and the anterior cerebral artery and might perfuse the posterior part of the rectal gyrus in addition to the anterior basal ganglia. Indeed, the recurrent artery of Heubner, as well as the subcallosal artery, is considered to be at high risk of injury during treatment of ACoA aneurysms [19].

It remains totally unclear why decreased rCBF in the subcallosal gyrus, anterior cingulate, and rectal gyrus only in the right hemisphere might be related to the memory impairment. A previous study reported that the resection or damage of the right rectal gyrus was associated with memory impairment [18]. Further studies with a larger population would be needed to confirm the right-hemispheric specialization in memory function.

Scoring of WMS-R is complex and might require experienced neuropsychologists. On the other hand, SPECT examination does not require specific proficiency, but provides objective evaluation of rCBF. It is generally used only in the acute or perioperative phase [20] to measure rCBF for evaluating the effect by vasospasm, but not often in the chronic phase. Considering the results in the current study, however, we believe that SPECT after the perioperative period could help to evaluate memory impairment in patients after ACoA aneurysm surgery.

The limitations of our study include the small number of patients and lack of a control group without symptoms of amnesia. Patients who had no memory impairment or only slight memory impairment after ACoA aneurysm surgery did not undergo SPECT due to the radiation exposure involved, and therefore, such subjects were not included in our study. However, the inclusion criteria selecting the patients with a specific impairment in memory might be the strength of our study, because our purpose was to clarify the relationship between SPECT findings and the degree of memory impairment. Further studies with larger subjects without or only slight memory impairment would be needed to confirm the current results. Another limitation is that we did not implement more detailed neuropsychological tests, including tests of confabulation, executive dysfunction, and personality change often found in ACoA syndrome. Such an analysis might be helpful for clarifying the cause of neuropsychological characteristics of ACoA syndrome, including memory impairment.

## Conclusion

The current study revealed the possibility that the decreased rCBF in the subcallosal gyrus, or basal forebrain, anterior cingulate gyrus, and rectal gyrus, especially in the right hemisphere, were related to postoperative amnesia.

## Electronic supplementary material

Below is the link to the electronic supplementary material.

Supplementary file1 (DOCX 50 kb)
