# Supplementary material for: Relationship between decreased cerebral blood flow and amnesia after microsurgery for anterior communicating artery aneurysm
Source: Ann Nucl Med. 2020 Jan 27;34(3):220–7. doi: 10.1007/s12149-020-01436-z (PMC7033071; doi:10.1007/s12149-020-01436-z)
Supplement: Supplementary file 1 — Supplementary file1 (DOCX 50 kb) [file 12149_2020_1436_MOESM1_ESM.docx]

**Supplemental Material**

**Relationship between decreased rCBF (*Severity and Extent*) and memory quotients (MQs)**

**(a) *Severity***

| **Bilateral Frontal lobe** | **Z score**  **(median,**  **(25, 75 percentile)** | **Verbal**  **MQ** | **Visual**  **MQ** | **General**  **MQ** |
| --- | --- | --- | --- | --- |
| Superior Frontal Gyrus R | 3.54  (3.27, 5.95) | ρ＝-0.648  p＝0.0425 | ρ＝-0.304  p＝0.391 | ρ＝-0.585  p＝0.0754 |
| Superior Frontal Gyrus L | 3.46  (2.78, 4.69) | ρ＝-0.115  p＝0.751 | ρ＝0.347  p＝0.325 | ρ＝0.061  p＝0.867 |
| Middle Frontal Gyrus R | 3.81  (2.80, 5.54) | ρ＝-0.648  p＝0.0425 | ρ＝-0.646  p＝0.0435 | ρ＝-0.713  p＝0.0205 |
| Middle Frontal Gyrus L | 3.28  (2.58, 4.00) | ρ＝-0.0304  p＝0.933 | ρ＝0.305  p＝0.390 | ρ＝0.0917  p＝0.801 |
| Inferior Frontal Gyrus R | 3.67  (2.94, 4.55) | ρ＝-0.563  p＝0.0897 | ρ＝-0.689  p＝0.0275 | ρ＝-0.701  p＝0.0239 |
| Inferior Frontal Gyrus L | 3.11  (2.36, 4.06) | ρ＝-0.218  p＝0.543 | ρ＝0.186  p＝0.605 | ρ＝-0.0856  p＝0.814 |
| Medial Frontal Gyrus R | 3.75  (3.09, 5.02) | ρ＝-0.697  p＝0.0251 | ρ＝-0.548  p＝0.100 | ρ＝-0.713  p＝0.0205 |
| Medial Frontal Gyrus L | 3.2  (2.57, 5.38) | ρ＝-0.272  p＝0.445 | ρ＝-0.0549  p＝0.880 | ρ＝-0.176  p＝0.625 |
| Orbital Gyrus R | 4.64  (2.91, 7.61) | ρ＝-0.636  p＝0.0479 | ρ＝-0.286  p＝0.422 | ρ＝-0.548  p＝0.100 |
| Orbital Gyrus L | 3.33  (1.93, 5.40) | ρ＝0.0851  p＝0.815 | ρ＝0.131  p＝0.717 | ρ＝0.113  p＝0.755 |
| Rectal Gyrus R | 5.35  (2.94, 7.22) | ρ＝-0.709  p＝0.0217 | ρ＝-0.701  p＝0.0239 | ρ＝-0.811  p＝0.0044* |
| Rectal Gyrus L | 2.83  (2.33, 4.83) | ρ＝-0.151  p＝0.676 | ρ＝0.213  p＝0.553 | ρ＝-0.0366  p＝0.920 |
| Paracentral Lobule R | 0  (0, 2.44) | ρ＝-0.320  p＝0.365 | ρ＝0.553  p＝0.0973 | ρ＝0.460  p＝0.180 |
| Paracentral Lobule L | 1.03  (0, 2.58) | ρ＝-0.653  p＝0.0407 | ρ＝0.696  p＝0.0254 | ρ＝0.728  p＝0.0169 |
| Precentral Gyrus R | 2.31  (0, 2.89) | ρ＝-0.206  p＝0.567 | ρ＝-0.125  p＝0.729 | ρ＝-0.210  p＝0.558 |
| Precentral Gyrus L | 1.03  (0, 2.57) | ρ＝-0.678  p＝0.0309 | ρ＝0.539  p＝0.107 | ρ＝0.683  p＝0.0295 |
| Subcallosal Gyrus R | 4.17  (3.61, 5.07) | ρ＝-0.721  p＝0.0186 | ρ＝-0.884  p＝0.0007* | ρ＝-0.853  p＝0.0017* |
| Subcallosal Gyrus L | 3.13  (1.81, 4.14) | ρ＝-0.182  p＝0.614 | ρ＝-0.305  p＝0.390 | ρ＝-0.238  p＝0.506 |
| **Bilateral Limbic lobe** |  |  |  |  |
| Fusiform Gyrus R | 2.9  (0, 3.51) | ρ＝-0.0061  p＝0.986 | ρ＝-0.129  p＝0.721 | ρ＝-0.0617  p＝0.865 |
| Fusiform Gyrus L | 1.03  (0, 2.63) | ρ＝0.0323  p＝0.929 | ρ＝-0.0065  p＝0.985 | ρ＝0.052  p＝0.886 |
| Lingual Gyrus R | 0  (0, 2.33) | ρ＝0.0224  p＝0.951 | ρ＝0.127  p＝0.725 | ρ＝0.112  p＝0.756 |
| Lingual Gyrus L | 0  (0, 0) | ρ＝-0.174  p＝0.630 | ρ＝0.175  p＝0.628 | ρ＝0  p＝1 |
| Thalamus R | 0  (0, 0) | NA  NA | NA  NA | NA  NA |
| Thalamus L | 0  (0, 0) | ρ＝0.406  p＝0.244 | ρ＝0.467  p＝0.173 | ρ＝0.467  p＝0.173 |
| Cingulate Gyrus R | 3.34  (3.11, 3.76) | ρ＝-0.345  p＝0.328 | ρ＝-0.176  p＝0.625 | ρ＝-0.329  p＝0.352 |
| Cingulate Gyrus L | 2.86  (2.64, 3.47) | ρ＝-0.345  p＝0.328 | ρ＝-0.0854  p＝0.814 | ρ＝-0.298  p＝0.401 |
| Parahippocampal Gyrus R | 2.75  (1.77, 3.24) | ρ＝0.297  p＝0.403 | ρ＝0.0642  p＝0.860 | ρ＝0.198  p＝0.582 |
| Parahippocampal Gyrus L | 2.38  (0, 2.73) | ρ＝-0.165  p＝0.647 | ρ＝-0.0494  p＝0.892 | ρ＝-0.0617  p＝0.865 |
| Anterior Cingulate R | 4.44  (3.84, 4.90) | ρ＝-0.769  p＝0.0092* | ρ＝-0.487  p＝0.152 | ρ＝-0.756  p＝0.0114 |
| Anterior Cingulate L | 4.07  (3.25, 4.44) | ρ＝-0.345  p＝0.328 | ρ＝-0.122  p＝0.737 | ρ＝-0.311  p＝0.381 |
| Posterior Cingulate R | 2.87  (1.63, 3.35) | ρ＝-0.0732  p＝0.840 | ρ＝-0.269  p＝0.450 | ρ＝-0.190  p＝0.598 |
| Posterior Cingulate L | 3.31  (2.79, 4.12) | ρ＝0.115  p＝0.751 | ρ＝0.207  p＝0.565 | ρ＝0.122  p＝0.737 |
| Uncus R | 2.59  (2.19, 3.87) | ρ＝-0.0545  p＝0.881 | ρ＝-0.426  p＝0.218 | ρ＝-0.170  p＝0.637 |
| Uncus L | 2.58  (0, 2.90) | ρ＝-0.500  p＝0.140 | ρ＝-0.446  p＝0.195 | ρ＝-0.493  p＝0.146 |
| **Right temporal lobe** |  |  |  |  |
| Superior Temporal Gyrus-Rt | 3.47  (2.69, 4.16) | ρ＝-0.466  p＝0.173 | ρ＝-0.524  p＝0.119 | ρ＝-0.536  p＝0.109 |
| Middle Temporal Gyrus-Rt | 3.34  (2.22, 3.83) | ρ＝-0.309  p＝0.384 | ρ＝-0.646  p＝0.0435 | ρ＝-0.493  p＝0.146 |
| Inferior Temporal Gyrus-Rt | 3.32  (2.85, 3.65) | ρ＝0.0545  p＝0.881 | ρ＝0.0854  p＝0.814 | ρ＝0.0854  p＝0.814 |
| Transverse Temporal Gyrus-Rt | 0  (0, 0.59) | ρ＝0.527  p＝0.117 | ρ＝0.343  p＝0.330 | ρ＝0.500  p＝0.140 |

R: Right

L: Left

NA: not applicable

*Statistically significant

**(b) *Exent***

| **Bilateral Frontal lobe** | **Extent (%)** | **Verbal**  **MQ** | **Visual**  **MQ** | **General**  **MQ** |
| --- | --- | --- | --- | --- |
| Superior Frontal Gyrus R | 44.3  (14.0, 53.6) | ρ＝-0.567  p＝0.0873 | ρ＝-0.567  p＝0.0873 | ρ＝-0.628  p＝0.0518 |
| Superior Frontal Gyrus L | 23.7  (13.5, 31.7) | ρ＝0.445  p＝0.197 | ρ＝0.445  p＝0.197 | ρ＝0.201  p＝0.577 |
| Middle Frontal Gyrus R | 20.6  (9.35, 36.9) | ρ＝-0.634  p＝0.0489 | ρ＝-0.634  p＝0.0489 | ρ＝-0.652  p＝0.0409 |
| Middle Frontal Gyrus L | 17.6  (5.35, 32.5) | ρ＝0.591  p＝0.0717 | ρ＝0.591  p＝0.0717 | ρ＝0.609  p＝0.0612 |
| Inferior Frontal Gyrus R | 23.7  (13.0, 46.9) | ρ＝-0.749  p＝0.0126 | ρ＝-0.749  p＝0.0126 | ρ＝-0.682  p＝0.0298 |
| Inferior Frontal Gyrus L | 13.7  (3.22, 38.0) | ρ＝0.474  p＝0.166 | ρ＝0.474  p＝0.166 | ρ＝0.174  p＝0.630 |
| Medial Frontal Gyrus R | 41.8  (15.2, 56.5) | ρ＝-0.554  p＝0.0959 | ρ＝-0.554  p＝0.0959 | ρ＝-0.493  p＝0.146 |
| Medial Frontal Gyrus L | 30.7  (12.5, 39.4) | ρ＝0.0795  p＝0.827 | ρ＝0.0795  p＝0.827 | ρ＝0.0031  p＝0.993 |
| Orbital Gyrus R | 100  (55.6, 100) | ρ＝-0.566  p＝0.0876 | ρ＝-0.566  p＝0.0876 | ρ＝-0.752  p＝0.0121 |
| Orbital Gyrus L | 63.6  (13.6, 100) | ρ＝0.0789  p＝0.828 | ρ＝0.0789  p＝0.828 | ρ＝0.138  p＝0.702 |
| Rectal Gyrus R | 87.5  (35.6, 100) | ρ＝-0.754  p＝0.0117 | ρ＝-0.754  p＝0.0117 | ρ＝-0.748  p＝0.0128 |
| Rectal Gyrus L | 49.2  (29.3, 81.2) | ρ＝0.128  p＝0.723 | ρ＝0.128  p＝0.723 | ρ＝-0.0153  p＝0.966 |
| Paracentral Lobule R | 0  (0, 18.8) | ρ＝0.606  p＝0.0628 | ρ＝0.606  p＝0.0628 | ρ＝0.565  p＝0.0884 |
| Paracentral Lobule L | 1.5  (0, 11.6) | ρ＝0.657  p＝0.039 | ρ＝0.657  p＝0.039 | ρ＝0.767  p＝0.0095 |
| Precentral Gyrus R | 3.4  (0, 10.9) | ρ＝-0.0063  p＝0.986 | ρ＝-0.0063  p＝0.986 | ρ＝-0.0472  p＝0.897 |
| Precentral Gyrus L | 0.2  (0, 5) | ρ＝0.715  p＝0.02 | ρ＝0.715  p＝0.02 | ρ＝0.741  p＝0.0141 |
| Subcallosal Gyrus R | 85  (45, 100) | ρ＝-0.770  p＝0.0091 | ρ＝-0.770  p＝0.0091* | ρ＝-0.683  p＝0.0294 |
| Subcallosal Gyrus L | 27.5  (11.2, 81.2) | ρ＝-0.3313  p＝0.3497 | ρ＝-0.3313  p＝0.3497 | ρ＝-0.398  p＝0.253 |
| **Bilateral Limbic lobe** |  |  |  |  |
| Fusiform Gyrus R | 24.725  (0, 17.85) | ρ＝0.0923  p＝0.7998 | ρ＝-0.0186  p＝0.9594 | ρ＝0.0619  p＝0.8651 |
| Fusiform Gyrus L | 8.975  (0, 0.55) | ρ＝0.1875  p＝0.604 | ρ＝0.1366  p＝0.7067 | ρ＝0.2212  p＝0.5392 |
| Lingual Gyrus R | 5.975  (0, 0) | ρ＝-0.0224  p＝0.9511 | ρ＝0.1425  p＝0.6944 | ρ＝0.09  p＝0.8047 |
| Lingual Gyrus L | 0  (0, 0) | ρ＝-0.1741  p＝0.6305 | ρ＝0.1751  p＝0.6284 | ρ＝0  p＝1 |
| Thalamus R | 0  (0, 0) | NA  NA | NA  NA | NA  NA. |
| Thalamus L | 0  (0, 0) | ρ＝0.4062  p＝0.2441 | ρ＝0.467  p＝0.1735 | ρ＝0.467  p＝0.1735 |
| Cingulate Gyrus R | 33.4  (19.75, 25.95) | ρ＝0.2242  p＝0.5334 | ρ＝-0.1768  p＝0.625 | ρ＝0.0366  p＝0.9201 |
| Cingulate Gyrus L | 35.475  (12.675, 22.1) | ρ＝0.2121  p＝0.5563 | ρ＝0.1707  p＝0.6372 | ρ＝0.1829  p＝0.613 |
| Parahippocampal Gyrus R | 60.175  (7.65, 23.45) | ρ＝-0.1829  p＝0.613 | ρ＝-0.2638  p＝0.4614 | ρ＝-0.2393  p＝0.5056 |
| Parahippocampal Gyrus L | 44.425  (0, 13.25) | ρ＝-0.2516  p＝0.4832 | ρ＝-0.2284  p＝0.5256 | ρ＝-0.1975  p＝0.5844 |
| Anterior Cingulate R | 91.825  (60, 80.55) | ρ＝-0.5515  p＝0.0984 | ρ＝-0.7744  p＝0.0085 | ρ＝-0.7317  p＝0.0162 |
| Anterior Cingulate L | 87.225  (52.25, 60) | ρ＝-0.4545  p＝0.1869 | ρ＝-0.4878  p＝0.1526 | ρ＝-0.5549  p＝0.0959 |
| Posterior Cingulate R | 16.1  (0.675, 5.45) | ρ＝-0.0304  p＝0.9336 | ρ＝-0.1162  p＝0.7492 | ρ＝-0.0703  p＝0.8469 |
| Posterior Cingulate L | 26.4  (9.775, 11.8) | ρ＝-0.1094  p＝0.7635 | ρ＝0.1835  p＝0.6119 | ρ＝0.0214  p＝0.9532 |
| Uncus R | 48.2  (2.1, 16.5) | ρ＝-0.2242  p＝0.5334 | ρ＝-0.4573  p＝0.1839 | ρ＝-0.2988  p＝0.4017 |
| Uncus L | 54.55  (0, 3.05) | ρ＝-0.4002  p＝0.2518 | ρ＝-0.5725  p＝0.0837 | ρ＝-0.4687  p＝0.1718 |
| **Right temporal lobe** |  |  |  |  |
| Superior Temporal Gyrus R | 17.15  (2.05, 31.05) | ρ＝-0.5273  p＝0.1173 | ρ＝-0.5488  p＝0.1004 | ρ＝-0.5976  p＝0.0681 |
| Superior Temporal Gyrus L | 3.9  (0.225, 15.675) | ρ＝0.3903  p＝0.2649 | ρ＝0.3067  p＝0.3886 | ρ＝0.4233  p＝0.2229 |
| Middle Temporal Gyrus R | 14.55  (1.2, 25.1) | ρ＝-0.3333  p＝0.3466 | ρ＝-0.3049  p＝0.3917 | ρ＝-0.3598  p＝0.3072 |
| Middle Temporal Gyrus  L | 0  (0, 2.85) | ρ＝-0.0956  p＝0.7928 | ρ＝-0.055  p＝0.8801 | ρ＝-0.0343  p＝0.925 |
| Inferior Temporal Gyrus  R | 16.55  (5.225, 36.025) | ρ＝-0.0788  p＝0.8287 | ρ＝-0.1159  p＝0.7499 | ρ＝-0.0854  p＝0.8146 |
| Inferior Temporal Gyrus  L | 0.3  (0, 4.35) | ρ＝0.0389  p＝0.915 | ρ＝-0.2056  p＝0.5688 | ρ＝-0.0196  p＝0.9572 |
| Transverse Temporal Gyrus R | 0  (0, 8.325) | ρ＝0.5276  p＝0.117 | ρ＝0.3438  p＝0.3308 | ρ＝0.5004  p＝0.1407 |
| Transverse Temporal Gyrus L | 0  (0, 0) | NA  NA | NA  NA | NA  NA |

R: Right

L: Left

NA: not applicable

*Statistically significant
